# Altered Brain Connectivity Patterns of Individual Differences in Insightful Problem Solving

**DOI:** 10.3389/fnbeh.2022.905806

**Published:** 2022-05-11

**Authors:** Jiabao Lin, Yajue Chen, Jiushu Xie, Lei Mo

**Affiliations:** ^1^Guangdong Key Laboratory of Mental Health and Cognitive Science, School of Psychology, Center for Studies of Psychological Application, South China Normal University, Guangzhou, China; ^2^Department of Psychology, School of Public Health and Management, Guangzhou University of Chinese Medicine, Guangzhou, China; ^3^Key Laboratory of Brain, Cognition and Education Sciences (South China Normal University), Ministry of Education, Guangzhou, China; ^4^Institut des Sciences Cognitives Marc Jeannerod, Université Claude Bernard Lyon 1, Lyon, France; ^5^School of Civil and Transportation Engineering, Guangdong University of Technology, Guangzhou, China; ^6^School of Psychology, Nanjing Normal University, Nanjing, China

**Keywords:** insightful problem solving, brain connectivity, resting-state fMRI, individual differences, neural mechanism

## Abstract

Insightful problem solving (IPS) attracts widespread attention in creative thinking domains. However, the neural underpinnings of individual differences in IPS are still unclear. The purpose of this research was to investigate inherent full-brain connectivity patterns at voxel-level in IPS. Sixty-two healthy participants were enrolled in the study. We used a voxelwise full-brain network measurement, degree centrality (DC), to depict the characteristics of cerebral network involved in individual differences in IPS. For each participant, we employed a chunk decomposition paradigm, using Mandarin characters as stimuli, to estimate the individual differences in IPS. Results showed that DC in the inferior frontal gyrus, and the middle frontal gyrus/precentral gyrus positively correlated with IPS, while the anterior cingulate cortex, and the brainstern/cerebellum/thalamus exhibited negative correlations with IPS. Using each cluster above as a seed, we performed seed-based functional connectivity analysis further. Results showed that IPS was mainly involved in the default mode network, containing the key regions of precuneus and medial prefrontal cortex. All in all, this research may shed new lights on understanding the neural underpinnings of individual differences in IPS.

## Introduction

Insightful problem solving (IPS) often denotes a new and sudden comprehension of the proper solution to a specific problem with positive feelings (i.e., Aha! experience). It has been suggested that IPS is associated with brain functions of set-shifting, mental restructuring, forming new associations, and emotion processing (Tik et al., 2018; Lin et al., 2020; Li et al., 2022). However, the neural substrates of individual difference in solving insightful problems remain elusive. As we know, brain underpinnings may precede behaviorally measurable cognitive processes. Uncovering sensitive brain areas might be advantageous for tracking the characteristics and cognitive processes of IPS. Furthermore, decoding the neural correlates of individual difference in IPS can elucidate why some individuals are inclined to have more insightful problem-solving success than others.

New brain imaging techniques, such as functional magnetic resonance imaging (fMRI), enable researchers to investigate neural substrates beneath the mental processes and individual differences in creative thinking. For instance, based on task-related fMRI, previous studies found that divergent thinking could involve the lateral prefrontal cortex, including ventrolateral and dorsolateral prefrontal cortex, which is considered as the neural mechanism of cognitive control (Benedek et al., 2018; Sun et al., 2019). The anterior and posterior cingulate cortex have frequently been discovered in divergent thinking studies (Adnan et al., 2019; Wu et al., 2020). Meanwhile, lateral and medial prefrontal cortex were also regarded as key brain regions in IPS, which could involve the processes of breaking mental sets, restructuring representations, and cognitive inhibition (Huang et al., 2015; Tang et al., 2015). Besides, hippocampal gyrus and amygdala were closely linked with IPS, since IPS recruited processes of forming novel associations and emotion encoding (Huang et al., 2015; Yu et al., 2019). We speculated that the lateral and medial prefrontal cortex might be the hub regions responsible for creative problem solving.

Our brain produces neural signals spontaneously and constantly even when no explicit tasks are performed (Biswal et al., 1995; Kong et al., 2015). These neural signals can help us uncover the relationship between resting-state brain activities, numerous cognitive processes, and psychological individual differences. Recently, resting-state (task-free) fMRI studies showed accumulated evidences about the neural correlates of creative thinking (Takeuchi et al., 2012; Wei et al., 2014; Wang et al., 2022). For example, studies regarding the relationship between individual difference of divergent thinking and brain spontaneous activities found that medial prefrontal and cingulate cortex play an important role in divergent thinking (Takeuchi et al., 2012). Similarly, another resting-state study focusing on creativity also revealed that medial prefrontal cortex was closely related to individual differences in creativity (Wei et al., 2014). These studies suggest strong linkage between spontaneous neural activity and creativity. However, as a typical creativity, insight’s relationship with the spontaneous neural activity has rarely been explored at the level of individual difference.

Resting-state fMRI researches have claimed brain mechanisms underlying creativity could be reflected from the perspective of functional brain network, and are not merely confined to regional alterations (Chen et al., 2014; Shi et al., 2018; Gao et al., 2021). For instance, a previous study found that the strength of resting-state functional connectivity (FC) between subcortical regions, such as thalamus and pallidum, can positively predict the degree of creative thinking (Gao et al., 2021). Chen et al. (2014) focused on individual differences in creativity, and revealed a negative relationship between the anterior cingulate (ACC) and prefrontal cortex in creativity. Besides, using an independent component analysis (ICA), Shi et al. (2018) found that anterior default mode network (DMN) correlating with frontal-parietal network contributes to divergent thinking. It’s worth noting that the above studies mainly depended on seed-based or ICA methods, aiming at the explorations within particular brain networks. The results derived from these analyses are diffusely distributed. Thus, a voxel-wise whole-brain FC analysis is urgently needed, which may provide novel insight into the brain underpinnings of creative thinking, especially for IPS.

Degree centrality (DC), a commonly used graph theory-based network measurement at voxel level, could estimate the significance of a specific region (i.e., node) in the whole-brain network. This method doesn’t need priori definition of regions of interest (ROIs), and can reveal details of the FC within the whole-brain network (Zuo et al., 2011). Many researches have confirmed DC indicator has physiological significance, reflecting features of brain blood flow and metabolism (Liang et al., 2013; Wang et al., 2021). Besides, DC measurements were proved to own good retest reliability (Zuo and Xing, 2014). Thus, researchers usually employed the DC method to depict characteristics of inherent brain networks underlying individual differences of cognitive process or psychiatric disorders (Di Martino et al., 2013; Sato et al., 2015; Li et al., 2020; Wang et al., 2021; Liu and Lai, 2022). Inspired by these studies, this research aimed to utilize the DC method to carry out a whole-brain detection of hub areas, showing changed brain connectivity responsible for individual differences in IPS. Finally, we used a seed-based approach to further examine the FC patterns of each cluster founded in DC analysis. Based on previous studies (Wei et al., 2014; Huang et al., 2015; Tang et al., 2015; Benedek et al., 2018; Lin et al., 2020), we predicted that hub regions, such as lateral or medial prefrontal cortex, may play a critical role in IPS, and IPS may be closely related to activations of the DMN.

## Materials and Methods

### Participants

Two criteria were used to recruit and select participants. Firstly, volunteers who signed up for the study were asked to fill out the Edinburgh Handedness Inventory to assess their hand preference. Only right-handed respondents were included as participants. Secondly, volunteers were estimated further according to their self-reports, and met the criteria: no history of psychiatric illness, no brain injury, normal vision, and no history of neurologic disease. As a result of these recruited requirements, sixty-two healthy native Mandarin Chinese speakers (28 males/34 females, aged 18–27 years old) were selected as participants with an average age of 20.93 (SD = 2.07). Notably, all volunteers were enrolled randomly, and have passed the university entrance examination. They were supposed to have similar cognitive abilities. This research got ethical approval from Research Ethics Review Board of South China Normal University. All volunteers signed a consent form before participation.

### The Assessment of Individual Differences in Insight Problem Solving

The research required participants firstly completed insight problem tasks, then received the magnetic resonance imaging (MRI) data acquisition. Specifically, individual differences of IPS were evaluated using the insightful Mandarin character chunk decomposition task, which consists of two tasks (the low insightful task and high insightful task). In the low insight task, participants removed a compound character from the given character to alter it into the target character. While in the high insight task, participants needed to remove an isolated character from the given character in order to obtain the target character. Previous studies have proved that the low and high insightful Mandarin character chunk decomposition tasks can successfully induce corresponding insight feelings, and details of the tasks can be found in these studies (Lin et al., 2019, 2020). Notably, the two insight tasks were together used to measure the individual differences of IPS. We firstly calculated the mean reaction time of both insight conditions. Secondly, the mean accuracy rates of the low and high insight conditions were computed. Finally, by dividing the mean reaction time into the mean accuracy rates, the inverse efficiency scores (ES) were obtained (Townsend and Ashby, 1978). Before analyzing, a log transform was performed on the inverse ES in order to address the skewness problem. There was a negative correlation between ES and individual differences of IPS. To put it another way, the lower the transformed ES values were, the better the insight performance. Upon completion of the behavioral tasks, all participants completed MRI scans, which consisted of structural scans (5 min), and resting-state scans (8 min).

### Magnetic Resonance Imaging Data Acquisition

Image collection was conducted with a 3.0 T Siemens Tim Trio MRI scanner with a 12-channel head coil at SCNU. A foam padding was provided to keep participant’s head still and therefore reduced head movements. Both the resting-state and structural imaging data were collected in this research. However, only resting-state data were further analyzed. The structural data were collected for the purpose of normalization. All resting-state images were determined by a gradient echo-planar imaging sequence. Parameters are showed below: Repetition time = 2000 ms; echo time = 30 ms; image thickness = 3.5 mm; field of view = 204 × 204 mm^2^; flip angle = 90°; data matrix = 64 × 64; 33 slices for a full brain. The brain anatomical images were acquired with a magnetization- radio-frequency pulses and rapid gradient-echo (MPRAGE) sequence. Parameters are showed below: Repetition time = 1900 ms; echo time = 2.52 ms; image thickness = 1.0 mm; flip angle = 9°; field of view = 256 × 256 mm^2^; data matrix = 256 × 256; 176 slices for a full brain.

### MRI Data Process

#### Data Preprocess

The image data were preprocessed and analyzed using the DPABI toolbox (Yan and Zang, 2010). First, in order to achieve scanner equilibration, the first ten images were removed. For the rest of 230 images, we further carried out a slice-timing correction and a head motion movement correction. It is worth noting that all the images fulfilled the head motion criteria: ≤ 2.5 mm translation; ≤ 2.5° rotation. Then, the volumes were co-registered with the structural ones, and standardized into a voxel size of 3 × 3 × 3 mm^3^, with a standard MNI template. Subsequently, all the volumes were further performed linear detrending and regressed out head motion parameters (computed by Friston 24-parameter model), white matter and cerebrospinal fluid signal. Meanwhile, mean framewise displacement (FD) of head motion was acquired and then employed as a factor of no interest to be regressed out in group-level analyses (Yan et al., 2013). Finally, temporal bandpass filtering (0.01–0.08 Hz) was conducted to minimize high-frequency physiological noise. Notably, in order to calculate the DC measurement, we didn’t perform smoothing for the images here considering previous studies (Zuo et al., 2011; Liu and Lai, 2022). Conversely, when evaluating the resting-state FC, all the volumes were smoothed with a 4 mm full-width at half-maximum (FWHM) resolution in this preprocessing stage.

#### Degree Centrality Analysis

The preprocessed images were tackled with the DPABI toolbox to get the DC values. DC is a parameter of the whole-brain network. It refers to the amounts of edges linking to a node. To calculate DC, we built a voxel-based whole-brain functional network. Each voxel was considered as a node. Then, we computed the Pearson’s correlation coefficients between each two voxels in the whole brain. The correlation coefficient is an indicator of the weight of connection, also known as “the edge.” We set a weight threshold of *r* > 0.25 (Buckner et al., 2009). Edges with an index ≤ 0.25 were discarded since it may be a correlation caused by random errors. Besides, edges with a negative coefficient were removed from the analysis. For each voxel, we calculated the DC (i.e., the sum of the threshold connections of each voxel), following these above-mentioned steps. We obtained a DC graph for each participant. All the DC graphs were then transformed using the Fisher-Z method for the purpose of normalization. Afterward, the normalized DC graphs were smoothed with a 4 mm FWHM resolution. In the end, a multiple regression model was performed to reveal the brain areas indicating prominent correlations between DC and individual differences of IPS (measured by ES). Notably, ES was set as the regressor of interest. Potential confounding factors (i.e., age, gender, and mean FD) were set as the regressors of no interest. Solving the multiple comparison problems, this research adopted a correction approach integrating voxel intensity (uncorrected *p* < 0.005) and cluster extent (FWE corrected *p* < 0.05).

#### Seed-Based Functional Connectivity Analysis

We used significant clusters found in the DC analysis as seed regions to compute FC alterations. For each seed, we estimated the mean time course within it, and then correlated it with the time course of other voxels within the gray matter mask. Afterward, the correlation coefficients were performed with Fisher’s r-to-z transformation to fulfill normality purposes, resulting in seed-based z-FC map for each participant. Finally, a multiple regression model was also performed to reveal the brain areas indicating prominent correlations between the FC and individual differences of IPS (measured by ES). Likewise, ES was set as the regressor of interest. Potential confounding factors (i.e., age, gender, and mean FD) were set as the regressors of no interest. Solving the multiple comparison problems, this research adopted a correction approach integrating voxel intensity (uncorrected *p* < 0.005) and cluster extent (FWE corrected *p* < 0.05).

## Results

### Behavioral Data

Mean (± SD) accuracies of the low insightful task and high insightful task were 0.94 ± 0.06 and 0.88 ± 0.07, respectively, and there was a significant difference between the two tasks (*t* = 7.59, *p* < 0.001). Mean (± SD) response times of the low insightful task and high insightful task were 1294.98 ± 481.30 and 1506.46 ± 553.98, respectively, and there was a significant difference between the two tasks (*t* = –7.64, *p* < 0.001). It’s worth noting that here we took response times with accuracies together to calculate the ES, a measurement reflecting individual difference of IPS. The ES indicated a normal distribution with a mean ± SD of 3.16 ± 0.17 in the present study.

### Degree Centrality

**FIGURE 1:**
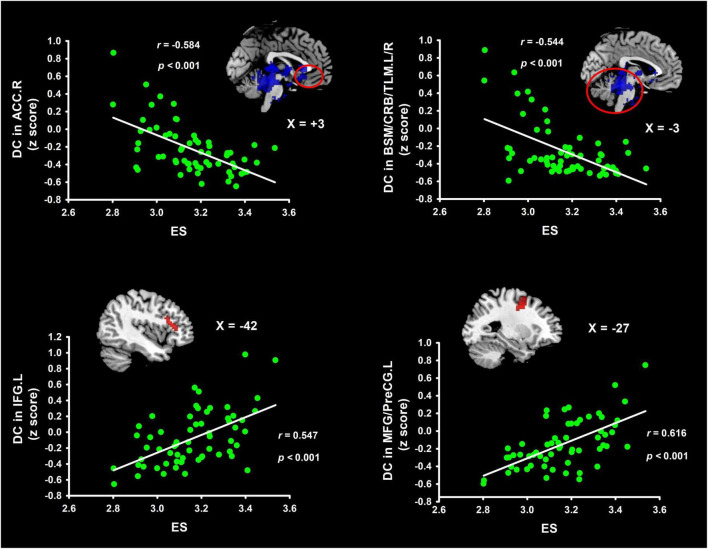
Clusters indicating correlations in weighted DC maps with ES. These brain clusters contain the IFG, MFG/PreCG, ACC, and BSM/CRB/TLM. Coordinates of Montreal Neurological Institute space were used in the present study. ES, efficiency score of insight; IFG, inferior frontal gyrus; TLM, thalamus; MFG, middle frontal gyrus; BSM, brainstern; PreCG, precentral gyrus; ACC, anterior cingulate cortex; CRB, cerebellum; DC degree centrality.

Table 1 and Figure 1 list the information on the clusters where there was a significant correlation between DC and ES. Detailly, ES correlated positively with DC, located at the left inferior frontal gyrus (IFG.L) and left middle frontal gyrus/precentral gyrus (MFG/PreCG.L). Besides, ES correlated negatively with DC in the right anterior cingulate cortex (ACC.R) and left/right brainstern/cerebellum/thalamus (BSM/CRB/TLM.L/R).

**TABLE 1:** Clusters indicating correlations between DC and ES.

Clusters	Side	Cluster size	MNI coordinates			*t* value
			
		(voxels)	*x*	*y*	*z*	
**Positive**						
IFG	L	75	–42	21	18	4.39
MFG/PreCG	L	86	–27	0	45	4.50
**Negative**						
ACC	R	83	3	21	3	–4.56
BSM/CRB/TLM	L, R	1318	–3	–33	–18	–5.09

*MNI, Montreal Neurological Institute; IFG, inferior frontal gyrus; BSM, brainstern; MFG, middle frontal gyrus; CRB, cerebellum; PreCG, precentral gyrus; ACC, anterior cingulate cortex; TLM, thalamus; ES, efficiency score of insight; DC, degree centrality; L, left; R, right. The t value refers to the statistical difference in the brain cluster.*

### Seed-Based Functional Connectivity

**FIGURE 2:**
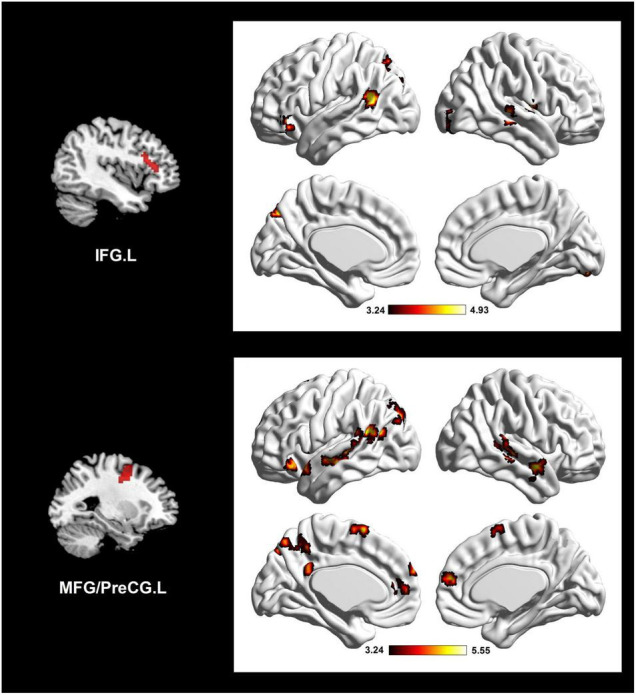
Altered network connectivity in the individual differences of insightful problem solving, using two seed clusters in the DC correlation analysis. Brain connectivity mainly involves ECN and DMN. The color bar refers to *t*-value. IFG.L, left inferior frontal gyrus; MFG.L, left middle frontal gyrus; PreCG.L, left precentral gyrus; ECN, executive control network; DMN, default mode network.

According to the results of DC analysis, four clusters were adopted as seed regions. We found sixteen significant ES-positive connections (Figure 2 and Table 2). Specifically, the strength of FC between the seed, IFG.L, and the right lingual gyrus (LING.R), IFG.L, right middle temporal gyrus (MTG.R), PreCG.R, left middle temporal gyrus (MTG.L), and left precuneus (PCUN.L) positively correlated with the ES. Besides, the strength of FC between the seed, MFG/PreCG.L, and the MTG.R, IFG.L, MTG.R, MTG.L, right superior temporal gyrus (STG.R), left/right medial prefrontal cortex (MPFC.L/R), MTG.L, PCUN.L, PCUN.L, and left/right supplementary motor area (SMA.L/R) also positively correlated with the ES. Notably, these ES-positive connections are mainly located in the DMN and executive control network (ECN).

**TABLE 2:** Brain regions showing correlations between the SBFC and ES. The *t* value refers to the statistical difference in a region.

Seed regions	SBFC regions	Cluster size	MNI coordinates			*t* value
			
		(voxels)	*x*	*y*	*z*	
IFG.L	LING.R	73	21	–84	–18	4.57
	IFG.L	50	–36	36	–9	4.61
	MTG.R	33	63	–27	–3	4.14
	PreCG.R	32	51	–6	15	4.60
	MTG.L	81	–57	–54	15	4.52
	PCUN.L	85	–21	–72	42	4.93
MFG/PreCG.L	MTG.R	43	54	–3	–15	5.14
	IFG.L	55	–54	15	–15	4.54
	MTG.R	52	60	–27	–3	4.43
	MTG.L	85	–54	–27	3	4.42
	STG.R	39	48	–36	12	4.69
	MPFC.L/R	67	6	54	18	4.37
	MTG.L	114	–39	–51	18	5.55
	PCUN.L	85	–3	–60	51	5.09
	PCUN.L	41	–21	–84	33	4.42
	SMA.L/R	37	0	6	63	4.98

*SBFC, seed-based functional connectivity; MNI, Montreal Neurological Institute; ES, efficiency score of insight; LING.R, right lingual gyrus; IFG.L, left inferior frontal gyrus; MTG.L, left middle temporal gyrus; MFG.L, left middle frontal gyrus; MTG.R, right middle temporal gyrus; PreCG.R, right precentral gyrus; PreCG.L, left precentral gyrus; PCUN.L, left precuneus; STG.R, right superior temporal gyrus; MPFC.L/R, left/right medial prefrontal cortex; SMA.L/R, left/right supplementary motor area.*

## Discussion

With graph theory-based approach, the present research has explored the brain connectivity patterns underlying individual differences of IPS, by integrating voxel-wise DC and seed-based FC methods. We found ES (an indicator reflecting individual difference of IPS) involved increased DC in the IFG.L and MFG/PreCG.L, while reduced DC in the ACC.R and BSM/CRB/TLM.L/R. Considering the inverse relationship between the ES and individual difference of IPS, IPS was thus related to reduced DC in the IFG.L and MFG/PreCG.L, suggesting network connections in these regions are reduced for IPS. Likewise, IPS was linked to increased DC in the ACC.R and BSM/CRB/TLM.L/R, implying network connections in these areas increase for IPS. Moreover, results of seed-based FC showed reduced connections from IFG.L and MFG/PreCG.L to dorsal/medial prefrontal cortex, lingual gyrus, temporal areas, and precuneus regions, mainly reflecting involvements of DMN and ECN in IPS.

Reduced DC and network connections in IPS were found in the IFG.L and MFG/PreCG.L. These brain regions are main parts of the lateral prefrontal cortex (Sasaki et al., 2018). The lateral prefrontal cortex, containing the dorsal lateral prefrontal cortex (DLPFC) and ventral lateral prefrontal cortex (VLPFC), mainly mediates cognitive control (Ochsner and Gross, 2005; Carballedo et al., 2011) and executive functions through IFG-MFG-striatum circuits (Quan et al., 2013). Besides, the DLPFC and VLPFC are also responsible for restraining dominant but disrelated responses and enabling cognitive flexibility (Derrfuss et al., 2005; Volle et al., 2011), which involves key components of IPS. Regarding the process of IPS, quite a lot of studies reported that IPS involves processes of inhibitory control and decision making, since certain cognitive control resources are required to overcome the mind set in IPS (Bowden, 1997; Knoblich et al., 1999; Lin et al., 2020). Furthermore, our findings were in accord with previous IPS researches (Gonen-Yaacovi et al., 2013; Zhao et al., 2014; Tik et al., 2018; Lin et al., 2020). For instance, using Chinese “chengyu” riddles as insight stimuli, Zhao et al. (2014) found that verbal insight problem solving had an altered FC anchored in the lateral prefrontal cortex than ordinary problem solving. Therefore, based on the roles of the DLPFC, VLPFC, and the processes of IPS reported in previous studies, we suggest that decreased connectivity in lateral prefrontal cortex could be critical for IPS.

Importantly, IFG.L and MFG/PreCG.L form the center of ECN, promoting cognitive and executive functions. Previous fMRI studies have identified changed ECN in IPS (Tang et al., 2015; Beaty et al., 2016; Huang et al., 2018). For instance, using chunk decomposition paradigm to investigate neural underpinnings under IPS with a parametric fMRI design, Tang et al. (2015) found that the ECN, such as DLPFC, determines optional ways of restructuring in IPS. In another study, regions in the ECN (e.g., LPFC) were found to negotiate novelty processing in IPS (Huang et al., 2018). Thus, altered connectivity of hub regions in ECN may represent a mechanism in IPS, in cases where many neural resources are required to select alternative ways of restructuring. The present study indicating a close relationship between ECN and individual differences of IPS may also support such an assumption.

Furthermore, seed-based FC analysis for IPS showed ECN anchored in the IFG.L and MFG/PreCG.L were also correlated with the hub regions of DMN, such as MPFC.L/R, MTG.L, STG.R, and PCUN.L. The DMN was supposed to facilitate automated information processing (Marron et al., 2018), and involve processes of self-monitoring, autobiographical memory retrieval, and mind wandering (Bressler and Menon, 2010; Andrews-Hanna, 2012; Zabelina and Andrews-Hanna, 2016). Accumulating evidence has showed that the DMN was related with creative thinking, including divergent thinking and IPS (Beaty et al., 2018; Fink et al., 2018; Marron et al., 2018; Lin et al., 2020; Wu and Chen, 2021). For example, Lin et al. (2020) found that activation in the DMN is a key signal discriminating the IPS from ordinary thinking. Moreover, several literatures indicated that both the DMN and ECN jointly support idea generation in creative performance (Beaty et al., 2016; Vatansever et al., 2017; Beaty et al., 2018). Vatansever et al. (2017) suggested when autonomous associative processing from DMN fails to reach unpredictable and novel target, external attention information will be required from the ECN. Likewise, Beaty et al. (2016) inferred that the DMN can help to the producing of candidate thoughts, while the ECN may estimate the efficacy of candidate thoughts and fulfill the goals of the task. They further suggested creative thinking might benefit from dynamic interactions of the DMN and ECN. Therefore, our present study indicating a function connectivity between ECN and DMN in IPS may provide more evidence for this hypothesis.

In the IPS, increased DC was detected in two other hub regions, the ACC.R and BSM/CRB/TLM.L/R. The ACC is mainly responsible for conflict control and detection (MacDonald et al., 2000; Anderson et al., 2009; Abe et al., 2018; Holroyd and Verguts, 2021). For instance, high conflict induces strong ACC activity when performing Stroop task (MacDonald et al., 2000). Several literatures have highlighted the ACC’s importance in IPS, and the ACC participated differentially in generating insight and non-insight solutions (Benedek et al., 2014; Shen et al., 2018a; Lin et al., 2020; Becker et al., 2021). In a review study, authors claimed that the BSM-thalamus-amygdala circuit plays a pivotal role in modulating and organizing emotion-related processing (Venkatraman et al., 2017). As we know, one striking feature of IPS is that individuals can experience positive emotions, such as the aha feelings (Luo and Niki, 2003; Shen et al., 2018b). We deduced that the BSM is related to the aha feelings in IPS. The TLM is the information relay station of the whole brain, where information was selected for further processing (Van Den Heuvel and Sporns, 2011; Boccia et al., 2015; Jung et al., 2015). Researchers suggested the TLM was linked to information filtering and cognitive control of the prefrontal cortex, and thus facilitated cognitive flexibility in creative thinking (Jung et al., 2015). Here, our study showing an increased connectivity in TLM may provide evidence for the role of cognitive flexibility in IPS. In addition, previous studies reported the CRB was responsible for cognitive control and executive functions, especially for the visual information (Keren-Happuch et al., 2014; Ogawa et al., 2018; Schmahmann et al., 2019). Specifically, Ogawa et al. (2018) proposed that restricting visual information can boost representational changes in creative insight. They further pointed out that the cerebellar networks coupled with the DMN may responsible for higher cognitive function, which could modulate the performance of IPS. Besides, a recent review confirmed that cerebellum acts as a hub critical for information detection, prediction and preparation, which subserves cognition. Intrinsic connectivity between the cerebellum network, the frontoparietal network, and the default network closely correlates with creativity and imagination (Schmahmann et al., 2019). Our results in this research are in accord with these previous literatures, supplying extra evidences that the ACC.R and BSM/CRB/TLM.L/R could involve individual differences of IPS.

### Limitations

Several limitations in this research should be tackled in future. First, increased and decreased connections can involve different physiological functions (Chelaru and Dragoi, 2008). For example, increased connectivity is linked with strengthen afferent information and facilitate signal selection. However, the meaning of increased and reduced connections in brain networks has not yet been clarified in the present study. Physiology researches will be required to tackle this issue in the future. Secondly, our FC in this study was non-directional. Other directed FC analyses, including Granger causality analyses, are urgently needed to provide direction information of the cortical networks in IPS. Finally, the present study focused on the individual differences of chunk decomposition, which is a special form of IPS. However, insight can vary differently. A previous study suggested that another crucial form of IPS, constraint relaxation, is worth further studying (Huang et al., 2018). Therefore, future studies should be conducted to reveal the neural basis of constraint relaxation in IPS, and to enrich insight theory and the understanding of individual differences in IPS.

## Conclusion

This study used a DC method, along with seed-based FC analysis, to explore the whole-brain intrinsic connectivity pattern underlying the individual differences of IPS. The results showed that the connectivity density is altered in four clusters (i.e., IFG.L, MFG/PreCG.L, ACC.R and BSM/CRB/TLM.L/R). Moreover, changed connectivity could be involved in the key nodes of DMN and ECN, which plays an important role in IPS. These results highlight the significance of brain connectivity in IPS, and might supply valuable information for the neural basis of individual differences in IPS.

## Data Availability Statement

The raw data supporting the conclusions of this article will be made available by the authors, without undue reservation.

## Ethics Statement

The studies involving human participants were reviewed and approved by Research Ethics Review Board of South China Normal University. The patients/participants provided their written informed consent to participate in this study.

## Author Contributions

JL and LM designed the study. JL and YC conducted data collection, data analyses, and interpretation. JL, JX, and YC drafted the manuscript. JL, JX, and LM provided critical revisions. All authors approved the final version of the manuscript for submission.

## Conflict of Interest

The authors declare that the research was conducted in the absence of any commercial or financial relationships that could be construed as a potential conflict of interest.

## Publisher’s Note

All claims expressed in this article are solely those of the authors and do not necessarily represent those of their affiliated organizations, or those of the publisher, the editors and the reviewers. Any product that may be evaluated in this article, or claim that may be made by its manufacturer, is not guaranteed or endorsed by the publisher.
